# Genetic Variants in the Protein S ( *PROS1* ) Gene and Protein S Deficiency in a Danish Population

**DOI:** 10.1055/s-0041-1736636

**Published:** 2021-10-28

**Authors:** Ole Halfdan Larsen, Alisa D. Kjaergaard, Anne-Mette Hvas, Peter H. Nissen

**Affiliations:** 1 Department of Molecular Medicine, Aarhus University Hospital, Aarhus, Denmark; 2 Department of Clinical Medicine, Aarhus University, Aarhus, Denmark; 3 Steno Diabetes Center Aarhus, Aarhus University Hospital, Aarhus, Denmark; 4 Department of Clinical Biochemistry, Aarhus University Hospital, Aarhus, Denmark

**Keywords:** protein S deficiency, *PROS1*, venous thromboembolism, inherited thrombophilia

## Abstract

Protein S (PS) deficiency is a risk factor for venous thromboembolism (VTE) and can be caused by variants of the gene encoding PS ( *PROS1* ). This study aimed to evaluate the clinical value of molecular analysis of the *PROS1* gene in PS-deficient participants. We performed Sanger sequencing of the coding region of the *PROS1* gene and multiplex ligation-dependent probe amplification to exclude large structural rearrangements. Free PS was measured by a particle-enhanced immunoassay, while PS activity was assessed by a clotting method.

A total of 87 PS-deficient participants and family members were included. In 22 index participants, we identified 13 *PROS1* coding variants. Five variants were novel. In 21 index participants, no coding sequence variants or structural rearrangements were identified. The free PS level was lower in index participants carrying a *PROS1* variant compared with index participants with no variant (0.51 [0.32–0.61] vs. 0.62 [0.57–0.73] × 10 ^3^ IU/L; *p* < 0.05). The p.(Thr78Met) variant was associated with only slightly decreased free PS levels (0.59 [0.53–0.66] × 10 ^3^ IU/L) compared with the p.(Glu390Lys) variant (0.27 [0.24–0.37] × 10 ^3^ IU/L, *p* < 0.01). The frequency of VTE in participants with a coding *PROS1* variant was 43 and 17% in the group with normal *PROS1* gene ( *p* = 0.05).

In conclusion, we report 13 *PROS1* coding variants including five novel variants. PS levels differ by *PROS1* variant and the frequency of VTE was higher when a coding *PROS1* variant was present. Hence, molecular analysis of the *PROS1* gene may add clinical value in the diagnostic work-up of PS deficiency.

## Introduction

Protein S (PS) is a vitamin K-dependent glycoprotein produced by the liver, which together with antithrombin and protein C constitutes the naturally occurring anticoagulation factors. PS serves as a cofactor for protein C, enhancing the proteolytic activity of protein C. In circulation, approximately 60% of PS is bound to the complement regulatory protein C4b-binding protein, while the remaining approximately 40% is circulated as free PS contributing to anticoagulation activity. The PS gene ( *PROS1* ) spans approximately 100 kb and consists of 15 translated exons that encode a 672 amino acid protein. 1

Hereditary PS deficiency is an autosomal-dominant condition caused by heterozygous variants in the *PROS1* gene (OMIM#612336). 2 3 Three types of PS deficiency exist: type I in which both PS level and activity are reduced. In type II PS deficiency, the circulating PS levels are normal, but the activity is reduced. Type III PS deficiency is characterized by total circulating PS in the normal range, while free PS and PS activity can be markedly reduced. 1 4

Venous thromboembolism (VTE) is a multifactorial condition comprising deep venous thrombosis and pulmonary embolism. VTE occurs as a result of environmental and genetic risk factors, such as inherited thrombophilia, e.g., caused by deficiencies in the naturally occurring anticoagulants. 5 In the general population, the prevalence of PS deficiency is estimated to be 0.03 to 0.1%, while the prevalence is estimated to be 2% in patients with VTE. 6 Recently, the allele frequency of *PROS1* variants putatively associated with PS deficiency was estimated to be 0.39% based on sequence data retrieved from the Exome Aggregation Database including more than 60,000 individual exomes. 7 Clinically, PS deficiency is associated with an increased risk of VTE 8 9 10 , and an increased risk of recurrent VTE. 8 9 11 However, in a recent meta-analysis, PS deficiency was shown not to be associated with recurrent VTE. 12

Diagnosing hereditary PS deficiency is complicated by the fact that numerous acquired conditions can cause temporary decreases in PS levels. These include decreased synthesis of PS due to, e.g., liver disease or anticoagulant treatments using vitamin K antagonists; PS consumption by, e.g., thrombosis, surgery, and disseminated intravascular coagulation, or redistribution of complexed PS in, e.g., pregnancy, and by use of oral contraceptives. 13 In addition, particularly the PS activity assays have the potential to generate false low PS values resulting in overdiagnosis of PS deficiency. 13 14 Hence, molecular genetic analyses of the *PROS1* gene may provide a helpful tool diagnosing hereditary PS deficiency.

The genetic spectrum of *PROS1* variants includes predominantly missense variants resulting in amino acid substitutions, but also comprises nonsense variants such as small insertions and deletions, splice-site variants, and large deletions spanning one or several exons. 8 15

In this study, our primary aim was to identify *PROS1* variants in PS-deficient participants, while the secondary aims were to explore any possible association with PS levels as well as thrombotic phenotype based on a systematic investigation of individuals from Danish families diagnosed with PS deficiency. The overall objective was to assess the diagnostic value and clinical use of molecular genetic analysis of the *PROS1* gene.

## Materials and Methods

### Participants

The study participants were recruited at the Thrombosis and Hemostasis Clinic at the Department of Clinical Biochemistry, Aarhus University Hospital. The individuals were identified by a systematic approach, using the electronic patient journal database, and it was applied to identify all patients registered with the diagnosis of PS deficiency at our department. To identify patients with contacts that predate the electronic system, the previous paper patient file system was reviewed manually. Identified PS-deficient patients were invited by letter to a new visit in the outpatient clinic and asked for potential participation in the study. In addition, newly referred participants suspected of PS deficiency in the study period were also invited to participate in the study. The ethical approval of the study did not allow identification and direct contact to the first-degree relatives of individuals with PS deficiency. Therefore, we informed all the participants that all their first-degree relatives were welcome to participate regardless of whether they had PS deficiency or not. At our center, a thrombophilia work-up is in general performed in patients below 50 years of age due to unexplained VTE as well as unexplained cerebral arterial thrombosis event (ATE) or peripheral ATE. Furthermore, family members to individuals with severe thrombophilia, e.g., deficiency of the natural anticoagulants, undergo thrombophilia investigations. Finally, women with pregnancy complications are examined and young women are tested before use of contraceptive pills if a family disposition to venous thromboembolic disease is apparent. Hence study participants were included based on thrombophilia work-up on an index patient by one of the following causes: (1) index patient with one or more VTE events, (2) index patient with one or more ATEs, (3) index patient with pregnancy complications in one or more cases (defined as intrauterine growth restriction/birth of a small-for-gestational-age neonate, preeclampsia, recurrent miscarriages, late pregnancy loss, or placental abruption), and (4) index patients with chance findings of low or borderline PS levels. PS deficiency was defined as two independent measurements of reduced free PS that could not be explained by temporary causes such as anticoagulants, pregnancy, or treatment with estrogens. The applied cut-off was the lower limit of the reference interval (0.69 × 10 ^3^ IU/L).At inclusion, a new diagnostic thrombophilia work-up was performed including PS measurements and molecular genetic analysis of the *PROS1* gene. Measurements of PS were only included if participants were either not receiving or were adequately paused in anticoagulant treatment known to affect PS levels. Furthermore, no pregnancy or treatment with estrogens was allowed for at least 3 months prior to blood sampling. Hence in some cases if the participant did not pause anticoagulant treatment at inclusion, PS data were extracted from medical records if PS values fulfilling the requirements were available within 1 year from the inclusion date.

Information on thromboembolic events and pregnancy complications was recorded based on systematic interview of the participants supported by medical records.

Eventually, we included 87 participants of which 70 had PS deficiency while five participants were included with inconclusive PS status. When possible, PS measurements fulfilling the requirements (see above) were obtained and evaluated in a total of 55 participants. Study participants were included from November 2015 to September 2018.

### Protein S Measurements

Free PS was measured on the Sysmex CS2100i coagulation system by a particle-enhanced immunoassay using the commercial INNOVANCE Free PS Ag kit (Siemens Healthineers, Erlangen, Germany). The reference interval was 0.69–1.37 × 10 ^3^ IU/L (locally determined 95% reference interval based on 105 blood donors). PS activity was measured on the ACL TOP 550 system by a clotting method using the commercial Hemosil Protein S Activity kit (Instrumentation Laboratory, Munich, Germany). The reference interval was 0.75–1.40 × 10 ^3^ IU/L (locally determined 95% reference interval based on 50 blood donors).

### Genetic Analysis

#### Polymerase Chain Reaction and Sequencing

DNA isolation was performed by use of either the QiaSymphony DSP mini kit (Qiagen, Hilden, Germany) or the Maxwell 16 Blood DNA Purification Kit (Promega, Nacka, Sweden) according to the manufacturer's instructions.

Protein coding exons and flanking intronic regions of the *PROS1* gene were amplified using previously reported primers. 15 The primers were modified with M13 linkers to facilitate sequencing. The polymerase chain reaction (PCR) products were purified by use of exonuclease and shrimp alkaline phosphatase digestion (ExoSAP-IT) as recommended by the manufacturer (Life Technologies Europe BV, Roskilde, Denmark). The purified fragments were bidirectionally sequenced using M13 sequencing primers (M13F: 5′ - GTAAAACGACGGCCAG – 3′ and M13R: 5′ – CAGGAAACAGCTATGAC – 3′) and BigDye terminator version 1.1 (Life Technologies). The sequencing reactions were ethanol-precipitated and separated on an Applied Biosystems 3500 or 3500xl Genetic Analyzer (Life Technologies). Sequence traces were aligned to NM_000313 ( *PROS1* ) by use of SeqScape software (version 2.7, Life Technologies).

Nomenclature of variants follows current guidelines. 16

#### Multiplex Ligation-Dependent Probe Amplification

Index participants were analyzed for large structural rearrangements, using multiplex ligation-dependent probe amplification (MLPA). We used the SALSA MLPA probemix P112-A3 PROS1 (MRC-Holland, Amsterdam, The Netherlands). Using this procedure, all *PROS1* exons, except exon 2 and exon 15, were targeted.

MLPA was performed essentially as described by the manufacturer. In brief, genomic DNA was denatured at 98°C for 5 minutes and hybridization of probes was performed by incubating each sample with the probe mix at 60°C for 16 hours. Following this, ligation proceeded at 54°C for 15 minutes. The resulting DNA with hybridized and ligated probes was amplified using the PCR primers supplied. The amplified fragments were separated on an Applied Biosystems 3500 or 3500xl Genetic Analyzer (Life Technologies) including the GS500ROX size standard (Life Technologies). The GeneMapper Software (version 4.1, Life Technologies) was used for visualization of fragment analysis data. MLPA profiles were assessed by visual inspection of the peaks.

##### Bioinformatics Analysis

We performed in silico assessment of missense variants using the prediction tools PolyPhen2, SIFT, and MutationTaster2. 17 18 19 Two intronic variants were assessed for possible activation of cryptic splice sites using the Splice Site Prediction tool 20 and the NetGene2 Server. 21 A putative promoter variant was assessed for possible disruption of promoter binding sites by the Neural Network Promoter Prediction tool. 22

Further, ClinVar ( https://www.ncbi.nlm.nih.gov/clinvar/ ), dbSNP ( https://www.ncbi.nlm.nih.gov/snp/ ), and gnomAD ( https://gnomad.broadinstitute.org/ ) were assessed for information on each specific variant.

##### Classification of Variants

The pathogenicity of the variants was evaluated using the classification system jointly proposed by the American College of Medical Genetics and Genomics and the Association for Molecular Pathology (ACMG-AMP). 23 This system classifies sequence variants in five classes as either pathogenic (class 5), likely pathogenic (class 4), uncertain significance (VUS, class 3), likely benign (class 2), or benign (class 1). The classification is based on a range of criteria, including the nature of the variant (e.g., nonsense or amino acid substitution), outcome of in silico predictions, segregation of the variant, and several other criteria. Assessment of each of these elements resulted in the assignment of a criterion in case the condition was fulfilled. All assigned criteria for each variant were evaluated using the ACMG-AMP as reference. All assigned criteria are provided in Supplementary Table S1 (online only).

### Statistics

The majority of the quantitative data did not follow a Gaussian distribution; thus all data were expressed as median with corresponding interquartile range (i.e., 25th and 75th percentiles) except for age where median with range was applied. Pairwise comparisons between two groups were performed using the Mann–Whitney test. For categorical data, Fisher's exact test was used. A probability ( *p* ) of 0.05 was used as the threshold for statistical significance.

Statistical analyses and figures were performed using GraphPad Prism version 8 (GraphPad Software, Inc., La Jolla, California, United States).

## Results

### Clinical Characteristics

Characteristics of the study population are shown in Table 1 . We included 75 participants of which 70 had PS deficiency while five participants were included with inconclusive PS status (borderline free PS results or contradictory PS measurements). Of these, 43 were index participants, presenting with VTE, ATE, pregnancy complications, or reduced PS levels, and 32 were PS-deficient relatives. In addition, 12 individuals, recruited as part of cascade screening in families with PS deficiency, had normal free PS levels and normal *PROS1* genotype.

**Table 1:** Characteristics of the study population

	Index participants	PS-deficient relatives	Healthy relatives
Participants, *N*	43	32	12
Women, *N* (%)	34 (79)	21 (66)	7 (58)
Age at inclusion ± SD, y	45 ± 17	45 ± 16	51 ± 12
ACT, *N* (%)	22 (51)	8 (25)	0 (0)
ACT in high-risk situations, *N* (%)	18 (42)	16 (50)	0 (0)
No ACT, *N* (%)	3 (7)	8 (25)	12 (100)

Abbreviation: ACT, anticoagulant treatment; PS, protein S; SD, standard deviation.

Note: Healthy relatives: relatives with normal free protein S levels.

### *PROS1* Variants and Classification

By a Sanger sequencing approach, we identified 16 different *PROS1* variants ( Table 2 ). Three variants were located in noncoding regions (5′ untranslated region [5′UTR] and exon flanking intronic regions). Ten were missense variants resulting in amino acid substitutions, while three were nonsense variants, either as a result of nucleotide substitutions resulting in a premature stop codon ( *N* = 2) or due to a 1 bp deletion resulting in a frameshift and a premature stop codon ( *N* = 1). Four variants were not previously reported but were present in dbSNP. Five variants were novel and neither published nor present in dbSNP, ClinVar, or gnomAD, while seven variants were previously published.

**Table 2:** Variants of the *PROS1* gene identified in Danish participants

*PROS1* region	Nucleotide change	Predicted amino acid change	dbSNP reference number	ACMG-AMP classification	References
Exon 1 (5′UTR)	c.-43G > A	–	rs370938580	Likely benign	Not reported
Exon 1	c.32T > C	p.(Leu11Pro)	NA	Uncertain significance	Novel
Intron 1	c.77–32A > G	–	rs778070336	Likely benign	Not reported
Exon 2	c.200A > C	p.(Glu67Ala)	rs766423432	Uncertain significance	Not reported
Exon 2	c.233C > T	p.(Thr78Met)	rs6122	Likely pathogenic	25 32 33 34
Exon 7	c.698G > A	p.(Arg233Lys)	rs41267007	Uncertain significance	35
Intron 8	c.728–20G > A	–	rs78230833 (G > A)	Likely benign	36
Exon 9	c.913C > T	p.(Gln305*)	rs1395378093	Pathogenic	33
Exon 10	c.992C > T	p.(Thr331Ile)	NA	Uncertain significance	Novel
Exon 10	c.1153A > G	p.(Met385Val)	rs766423432	Uncertain significance	Not reported
Exon 11	c.1168G > A	p.(Glu390Lys)	NA	Likely pathogenic	37 38
Exon 11	c.1241T > C	p.(Phe414Ser)	NA	Uncertain significance	Novel
Exon 12	c.1351C > T	p.(Arg451*)	rs5017717	Pathogenic	33 37
Exon 12	c.1468del	p.(Ile490Leufs*6)	NA	Pathogenic	Novel
Exon 13	c.1501T > C	p.(Ser501Pro)	rs121918472	Uncertain significance	37 39
Exon 13	c.1577T > C	p.(Leu526Ser)	NA	Likely pathogenic	Novel

Abbreviation: dbSNP, Single Nucleotide Polymorphism Database.

Using the ACMG-AMP criteria for variant classification, 23 three variants were classified as likely pathogenic (class 4), while the three nonsense variants were classified as pathogenic (class 5). We classified seven of the variants as VUS (class 3). This classification is due to several factors, e.g., lack of family members to follow segregation of the variant with PS deficiency and disagreement of functional consequence between in silico prediction methods. The three noncoding variants were classified as class 2, likely benign variants ( Supplementary Table S1 [online only]).

To identify potential large complex rearrangements of the *PROS1* gene, such as deletions spanning one or more exons, MLPA was performed in all index participants (two samples failed due to technical reasons). We did not identify large rearrangements of the *PROS1* gene in any participants in this study.

### Protein S Levels Differ by *PROS1* Variant

**Fig. 1:**
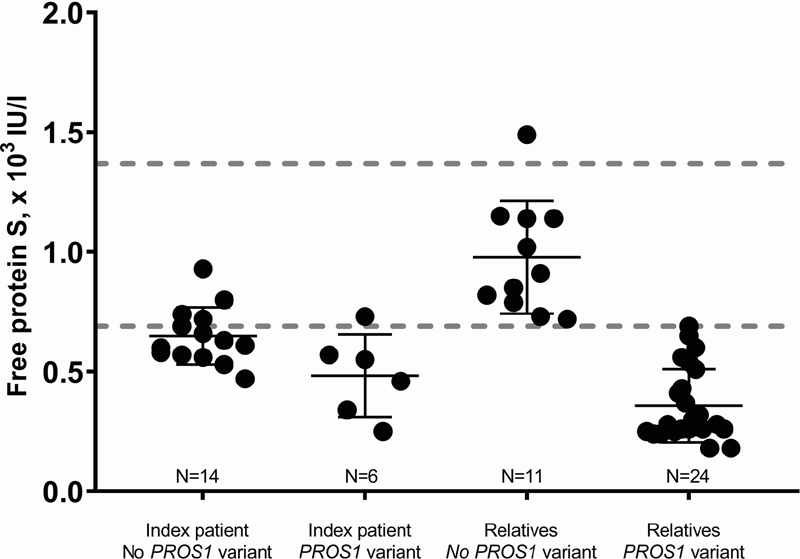
Free protein S levels in protein S-deficient index participants with a *PROS1* variant or with normal *PROS1* sequence and relatives with *PROS1* variant or normal *PROS1* sequence. *PROS1* variants included were: p.(Glu67Ala), p.(Thr78Met), p.(Gln305*), p.(Thr331Ile), p.(Met385Val), p.(Glu390Lys), p.(Arg451*), p.(Ile490Leufs*6), p.(Ser501Pro), p.(Leu526Ser). The reference interval for free protein S (0.69–1.37 × 10 ^3^ IU/L) is indicated by *dotted horizontal lines* .

We evaluated PS levels, and found that the free PS level was lower in index participants carrying a coding *PROS1* variant compared with index participants with no *PROS1* variant (0.51 [0.32–0.61] × 10 ^3^ IU/L ( *N* = 6) vs. 0.62 [0.57–0.73] × 10 ^3^ IU/L ( *N* = 14); *p* = 0.03; Fig. 1 ). One exception was a participant double heterozygous for the p.(Thr78Met) and the p.(Arg233Lys) variants presenting with a free PS of 0.73 ( Fig. 1 ). As expected, the control group consisting of individuals included as part of family screening, with no *PROS1* variants, had free PS levels in the normal range ( *N* = 11, data from one individual missing, Fig. 1 ).

In the available cases, there were no difference between free PS and PS activity for participants with no *PROS1* variant (0.62 [0.57–0.73] × 10 ^3^ IU/L ( *N* = 14) vs. (0.65 [0.51–0.76] × 10 ^3^ IU/L ( *N* = 10), *p* = 0.99). Likewise, no difference was observed in free PS and PS activity for participants with a *PROS1* variant (0.51 [0.32–0.61] × 10 ^3^ IU/L ( *N* = 6) vs. (0.51 [0.38–0.62] × 10 ^3^ IU/L ( *N* = 3), *p* = 0.90).

**Fig. 2:**
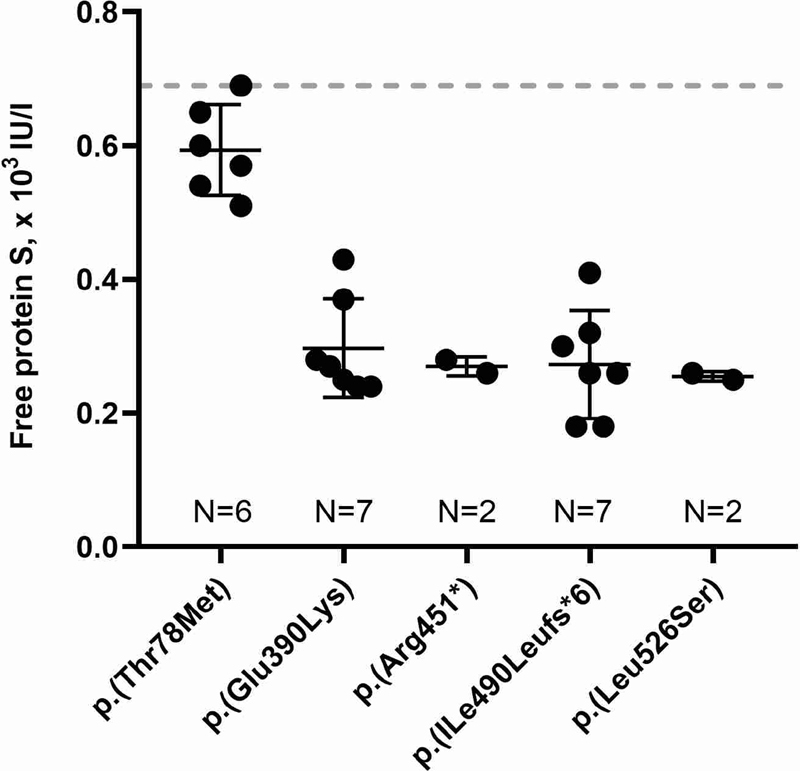
Free protein S levels in protein S-deficient participants carrying five specific *PROS1* variants. The lower reference limit for free protein S (0.69 × 10 ^3^ IU/L) is indicated by a *dotted horizontal line* .

To explore genotype–phenotype correlations, free PS levels were grouped by variant in cases where data on two or more individuals were available. Fig. 2 indicates that the p.(Thr78Met) variant resulted in only slightly decreased free PS levels (0.59 [0.53–0.66] × 10 ^3^ IU/L, *N* = 6) compared with, e.g., the p.(Glu390Lys) variant (0.27 [0.24–0.37] × 10 ^3^ IU/L, *N* = 7, *p* = 0.001). One participant included during family screening and carrying the p.(Thr78Met) had free PS level of 0.69 × 10 ^3^ IU/L corresponding to the lower reference limit.

**Fig. 3:**
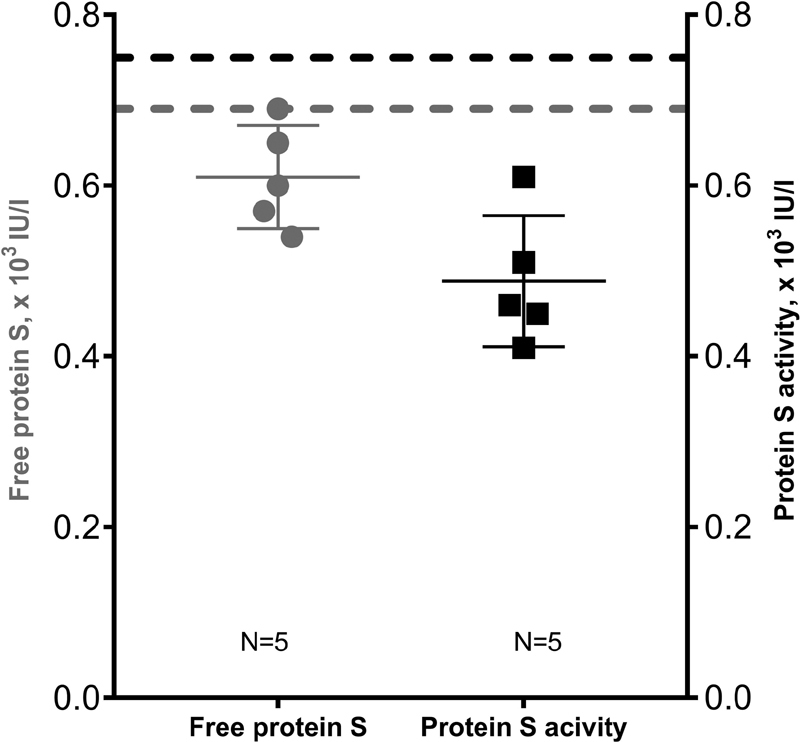
Free protein S and protein S activity for participants heterozygous for the p.(Thr78Met) variant of the *PROS1* gene. *Gray dots* represent participants with free protein S values ( *N* = 5). The *gray broken line* indicates the lower reference limit (0.69 × 10 ^3^ IU/L) of the free protein S assay. Protein S activity measurements were available for the same five, presented as *black squares* with the corresponding lower reference limit of 0.75 × 10 ^3^ IU/L in the *black broken line* .

To further evaluate if the reduced free PS levels, associated to the p.(Thr78Met) variant, might be due to interference in the free PS assay, rather than representing a quantitative defect, we compared the free PS and PS activity levels for this variant ( Fig. 3 ). The median level of the PS activity measurements was 0.46 [0.43–0.56] × 10 ^3^ IU/L ( *N* = 5, reference range: 0.69–1.37 × 10 ^3^ IU/L) compared with 0.60 [0.56–0.69] × 10 ^3^ IU/L ( *N* = 5, reference range: 0.75–1.40 × 10 ^3^ IU/L) for the free PS assay ( *p* = 0.06).

### Reason for Referral and *PROS1* Variant Detection

We identified a total of 23 coding variants in 43 index participants, resulting in a crude detection rate of 53%.

To investigate the detection rate of the molecular genetic analysis in a clinical setting, different scenarios were established based on the reason for referral. Hence, we analyzed the detection rate in the subsets of index participants where the reason for referral was one of the three major indications: (1) a personal history of VTE ( *N* = 18), (2) ATE ( *N* = 9), or (3) pregnancy complications ( *N* = 4).

In 83% (15 of 18) of the index participants referred due to VTE, a coding *PROS1* variant was identified. For index participants referred due to ATE the detection rate was 11% (1 of 9). When the reason for referral was pregnancy complications, the detection rate was 25% (1 of 4).

### *PROS1* Variants and Venous Thromboembolism

The clinical characteristics of PS-deficient participants are summarized in Tables 3 and 4 . Of 18 index participants with no detectable *PROS1* variants, three participants experienced VTE (17%). Of these, one participant had two events at the age of 15 and 24 years. A total of 54 participants carried a coding *PROS1* variant. Of these, 43% experienced one or more VTEs. Testing the hypothesis that VTE is independent of having a variant in the *PROS1* gene was just rejected by Fisher's exact test at the 5% significance level ( *p* = 0.05). Thus, our data cannot rule out, that the apparent overweight of participants with VTE, which were heterozygous for a *PROS1* variant, was a chance finding.

**Table 3:** Clinical characteristics of 75 study participants (43 index participants and 32 family members)

Nucleotide change	Predicted amino acid change	Participants, *N* (families)	VTE, *N* (%)	Recurrent VTE, *N* (%)	Index participants with family history of VTE, *N* (%)	ATE, *N* (%)	Recurrent ATE, *N* (%)	Pregnancy complications, *N* (% of women)
No variant detected	–	18 (18)	3 (17)	1 (6)	4 (22)	7 (39)	1 (6)	6 (33)
c.-43G > A	–	2 (1)	0 (0)	0 (0)	1 (100)	0 (0)	0 (0)	0 (0)
c.32T > C	p.(Leu11Pro)	1 (1)	1 (100)	1 (100)	1 (100)	0 (0)	0 (0)	0 (0)
c.77–32A > G	–	1 (1)	1 (100)	0 (0)	0 (0)	0 (0)	0 (0)	0 (0)
c.200A > C	p.(Glu67Ala)	2 (2)	2 (100)	0 (0)	1 (50)	0 (0)	0 (0)	0 (0)
c.233C > T	p.(Thr78Met)	9 (3)	0 (0)	0 (0)	2 (22)	2 (22)	0 (0)	1 (13)
c.233C > T; c.698G > A	p.(Thr78Met); p.(Arg233Lys)	1 (1)	1 (100)	0 (0)	1(100)	0 (0)	0 (0)	0 (0)
c.728–20G > A	–	1 (1)	0 (0)	0 (0)	1 (100)	1 (100)	0 (0)	0 (0)
c.913C > T	p.(Gln305*)	1 (1)	0 (0)	0 (0)	1 (100)	0 (0)	0 (0)	0 (0)
c.992C > T	p.(Thr331Ile)	1 (1)	0 (0)	0 (0)	1 (100)	0 (0)	0 (0)	0 (0)
c.1153A > G	p.(Met385Val)	1 (1)	0 (0)	0 (0)	1 (100)	0 (0)	0 (0)	1 (100)
c.1168G > A	p.(Glu390Lys)	15 (6)	9 (56)	5 (31)	5 (83)	1 (6)	0 (0)	1 (13)
c.1241T > C	p.(Phe414Ser)	1 (1)	1 (100)	0 (0)	0 (0)	0 (0)	0 (0)	0 (0)
c.1351C > T	p.(Arg451*)	3 (1)	1 (33)	1 (33)	0 (0)	1 (33)	1 (33)	0 (0)
c.1468del	p.(Ile490Leufs*6)	12 (1)	5 (42)	4 (33)	1 (100)	1 (8)	0 (0)	0 (0)
c.1501T > C	p.(Ser501Pro)	2 (2)	2 (100)	1 (50)	0 (0)	0 (0)	0 (0)	0 (0)
c.1577T > C	p.(Leu526Ser)	4 (1)	2 (50)	0 (0)	1 (100)	0 (0)	0 (0)	2 (100)

Abbreviations: ATE, arterial thrombosis event; VTE, venous thromboembolism.

**Table 4:** Thromboembolic events and pregnancy by variant status ( *PROS1* variants class 3–5)

	*PROS1* coding variant	Normal *PROS1* gene	*p* -Value
Total number of participants	54	18	
Women	35 (65)	18 (100)	0.32
Age at inclusion in years	45 (19–80)	35 (20–60)	0.02
Age at first venous thromboembolic event	30 (14–64)	21 (15–27)	0.09
Venous thromboembolic events	23 (43)	3 (17)	0.05
Deep vein thrombosis	12 (52)	3 (100)	
Pulmonary embolism	4 (17)	0 (0)	
Deep vein thrombosis and pulmonary embolism	5 (22)	0 (0)	
Other (vena porta or retinal vein thrombosis)	2 (9)	0 (0)	
Recurrent venous thromboembolic events	12 (52)	1 (33)	
Arterial thromboembolism	6 (11)	7 (39)	0.01
Recurrent arterial thromboembolic events	1 (2)	1 (6)	
Pregnancy complications	5 (9)	6 (33)	0.02
Recurrent pregnancy complications	4 (7)	2 (11)	

Note: Values are *N* (%) or median (range).

### Arterial Thromboembolism and Pregnancy Complications

Arterial thromboembolism was more frequent in the group of participants with normal *PROS1* gene compared with participants with a *PROS1* variant (39 vs. 11%, *p* = 0.01). Recurrent ATE was reported in one case in both groups of Table 4 .

A range of pregnancy complications was reported: abruptio placentae, preeclampsia, early abortion, spontaneous abortion, intrauterine fetal death, and premature birth. Pregnancy complications were reported more frequent in the group of participants with normal *PROS1* gene compared with the group with a *PROS1* variant (33 vs. 9%, *p* = 0.02, Table 4 ). However, recurrent pregnancy complications were reported in four of the five cases in the group of *PROS1* variant carriers and in two of six cases in the group with normal *PROS1* gene ( Table 4 ).

## Discussion

Since the heritable nature of PS deficiency was first acknowledged as a contributing factor to the development of VTE, 2 3 considerable progress has been made in understanding the contribution of genetic variants of the *PROS1* gene to the disease outcomes of PS deficiency. However, it is unclear whether molecular genetic analysis of the *PROS1* gene provides further clinical insights into the diagnostic work-up of PS deficiency, and in a recent guideline, the clinical value of genetic analysis of *PROS1* is not directly addressed. 14

In this study, we performed molecular genetic analysis of participants with PS deficiency and identified 16 different *PROS1* variants of which nine were not previously reported as variants associated with PS deficiency. These results expand the spectrum of *PROS1* variants associated with PS deficiency and support other studies showing heterogeneity in the genetic background of PS deficiency. 15 24 25

We classified the variants using the ACMG-AMP criteria. 23 Seven variants were classified as VUS making these a challenge in terms of clinical action. In the present study two factors were predominantly decisive in a classification as a VUS: disagreement in the consequence of the variant predicted by in silico prediction tools and lack of information on segregation of the variant. The first is a well-known problem in prediction tools, each tool having different ability to correctly assess the functional consequence of the variants. In the ACMG-AMP criteria, multiple lines of computational evidence supporting a deleterious effect of a variant weigh as supporting evidence of pathogenicity. However, as reviewed by Masica and Karchin, PolyPhen2 and SIFT, two of the prediction tools used in the present study, have a prediction accuracy ranging from 62 to 80% dependent on the dataset assessed. 26 As a consequence, the ACMG-AMP classifier related to computational evidence may often be the decisive classification element tipping a variant from likely pathogenic to a VUS.

Large deletions of the *PROS1* gene have been associated with PS deficiency in individuals where no other *PROS1* variants were identified; however, the extent of this type of variant seems to vary a great deal. Caspers and coauthors identified a large deletion in five of 185 individuals, 15 while a smaller study identified a *PROS1* deletion in 33% of PS-deficient probands. 27 In the present study, we did not identify large complex rearrangements of the *PROS1* gene by use of MLPA.

The c.-43G > A variant, located in the 5′UTR, was of interest since a recent study identified a c.-39C > T variant that was shown in vitro to introduce a new translation initiation codon, and consequently a premature stop codon and suggested to cause PS deficiency. 28 However, in the present study, one family member heterozygous for the c.-43G > A variant had normal PS levels, indicating that this is a variant with no impact on the PS phenotype.

We observed statistically significantly lower PS levels in index participants heterozygous for a *PROS1* variant than in index participants with normal *PROS1* gene. In addition, our data suggest that protein coding variants are associated with variable phenotypes of PS plasma levels, particularly driven by the observations on the p.(Thr78Met) variant. Participants heterozygous for the p.(Thr78Met) variant had higher free PS levels than participants heterozygous for, e.g., the p.(Glu390Lys) variant ( *p* = 0.001, Fig. 2 ). To rule out that the p.(Thr78Met) variant was not a normal variant interfering with the free PS assay, resulting in false low free PS values, we compared free PS results to PS activity in available cases with both measurements. There was no statistically significant difference between PS activity values and free PS values, indicating that the p.(Thr78Met) is a variant that causes only slightly reduced PS levels. Further, none of the nine participants heterozygous for the p.(Thr78Met) variant presented with VTE. However, two presented with ATE and one experienced pregnancy complications. To our knowledge, this is the first study to show genotype–phenotype correlation on the variant level.

The p.(Glu390Lys) was the most frequent variant identified in 26% of the index participants in the present study group. In an early study of Danish PS-deficient families, it was suggested based on microsatellite and haplotype analysis that the p.(Glu390Lys) variant was a founder variant. This is consistent with the high frequency of this variant among PS-deficient index participants in our study. If this is also the case for the p.(Thr78Met) variant, which was present in 17% of the index participants, remains to be investigated.

Apart from several case reports, a few studies of cohorts of similar size to the present study have reported on the association between *PROS1* variants and clinical manifestations such as VTE. 24 25 We observed a higher frequency (43%) of PS-deficient participants with VTE carrying a *PROS1* variant than in the group not carrying a *PROS1* variant (17%); however, the data were not statistically significant ( *p* = 0.05). In a study of cases of unprovoked idiopathic fatal pulmonary embolism, it was shown by a whole exome sequencing approach that the risk of death was highly increased in cases heterozygous for a *PROS1* variant compared with the control group (odds ratio = 56.4, *p* = 0.001). 29

In our cohort the clinical data on arterial thromboembolic events and pregnancy complications suggest an opposite dependency compared with VTE. For both ATE and pregnancy complications, the frequency of events was higher in the groups with no *PROS1* variant compared with PS-deficient participants heterozygous for a *PROS1* variant ( *p* = 0.02). However, recurrent pregnancy complications were more frequent in the group of *PROS1* gene variant carriers.

In the present study, the crude detection rate of a coding *PROS1* variant was 52%, while the detection rate increased substantially to 83% for index participants referred due to VTE. Caspers and colleagues showed a variant detection rate of 43% with an inclusion criterion of participants with a personal history of a thromboembolic event in association with reduced PS activity levels. 15 In the opposite end of the variant detection spectrum, the study by Ten Kate and colleagues identified a *PROS1* variant in 35 of 36 PS-deficient probands, corresponding to a detection rate of 97%. 30 Contrary to this, we observed low detection rates of 11 and 25% when the reason for referral was ATE and pregnancy complications, respectively. The large spectrum of variant detection rates and our results show that the establishment of PS deficiency and a personal history of VTE increase the likelihood of identifying a *PROS1* variant.

These findings together, bearing the challenges in the plasma assays for PS in mind, suggest that *PROS1* genotyping may be a useful tool in the diagnostic work-up of PS deficiency particularly for VTE. When the reason for referral is ATE and pregnancy complications, the value of molecular genetic analysis of the *PROS1* gene is more questionable.

Further, our study suggests that it may be possible to stratify treatment based on genotype, since carriers of the p.(Thr78Met) variant seem to be less prone to VTE than carriers of, e.g., the p.(Glu390Lys) variant. However, this strategy needs to be confirmed in future clinical studies, and the present study does not allow us to conclude causal effects.

The present study represents the largest cohort of Danish PS-deficient participants thoroughly studied by molecular genetic analysis. Only few studies have reported systematically on the association between *PROS1* genotype and thromboembolic events. Thus, our data represent a step forward and an opportunity to improve the diagnostic work-up of patients with PS deficiency. Some limitations of the study need consideration.

As the study participants were recruited based on medical records, selection bias must be considered as index participants were included based not only on decreased PS levels but also on thrombotic events. This makes it possible that decreased PS levels may be chance findings with no causal effect. Further, it was not possible to perform follow-up, thus we cannot reject that some study participants have developed events later in life. We measured free PS as standard care and PS activity in several cases. However, total PS was not measured in this study, limiting the ability to distinguish between type I and type III PS deficiency. The use of total PS measurements is not common practice as it will not contribute significantly to the final diagnosis, 13 and distinguishing between type I and type III PS deficiency will rarely impact the treatment or counseling of the patient and family. In our study, all the 18 participants without a *PROS1* variant were women. Previous studies have indicated that the PS levels are lower in women than in men. 31 Therefore, it is possible that these women may not have genetically determined PS deficiency, which is supported by the genetic data and lack of *PROS1* variants. The association of thromboembolic events and *PROS1* variant relies on small groups. Increasing the number of participants might have revealed more reliable associations. Finally, the evaluation of the variants could have been improved by performing functional in vitro studies of novel variants. To compensate for this limitation, we performed a stringent classification based on current ACMG guidelines.

In conclusion, this study expands the spectrum of genetic variants in the *PROS1* gene associated with PS deficiency and reports a genotype–phenotype correlation on the variant level. A total of 16 different *PROS1* variants were detected, of which 13 were classified as pathogenic, likely pathogenic, or VUS. We observed that the p.(Thr78Met) variant in the present dataset was associated with higher PS levels than other *PROS1* variants. In participants with reduced PS levels, the frequency of VTE was higher when a coding *PROS1* variant was present. These data suggest that molecular analysis of the *PROS1* gene may provide clinical value in the diagnostic work-up of PS deficiency, including the potential for improved risk prediction for development of VTE.
