# Supplementary material for: Genetic Variants in the Protein S ( PROS1 ) Gene and Protein S Deficiency in a Danish Population
Source: TH Open. 2021 Oct 28;5(4):e479–88. doi: 10.1055/s-0041-1736636 (PMC8553426; doi:10.1055/s-0041-1736636)
Supplement: Supplementary file 1 — Supplementary Material [file 10-1055-s-0041-1736636-s210032.pdf]

Supplementary Table S1 *PROS1* variants in Danish protein S-deficient individuals: in silico predictions, database information, ACMG-AMP classification, and references

Region	Nucleotide change	Predicted amino acid change	PolyPhen2 HumVar prediction	SIFT prediction	Mutation Taster prediction	dbSNP reference number	ClinVar interpretation	GnomAD allele frequency (*N* homozygotes)	ACMG criteria for classification	ACMG classification	References
Exon 1 (5′UTR)	c.-43G > A	–	NA	NA	NA	rs370938580	Uncertain significance	9.73e-5 (0)	PM2, PP4, BS2	Likely benign	Not reported
Exon 1	c.32T > C	p.(Leu11Pro)	Probably damaging	Affect protein function	Disease causing	NA	NA	NA	PM2, PP2, PP3, PP4	Uncertain significance	Novel
Intron 1	c.77–32A > G	–	NA	NA	NA	rs778070336	NA	1.05e-4 (0)	PP4, BP7	Likely benign	Not reported
Exon 2	c.200A > C	p.(Glu67Ala)	Probably damaging	Affect protein function	Disease causing	rs766423432	Likely pathogenic	3.89e-5 (0)	PM2, PP2, PP3, PP4	Uncertain significance	Not reported
Exon 2	c.233C > T	p.(Thr78Met)	Probably damaging	Affect protein function	Disease causing	rs6122	Conflicting interpretations of pathogenicity	5.17e-5 (0)	PM2, PP1, PP2, PP3, PP4, PP5	Likely pathogenic	Gandrille S et al. Blood. 1995 Jan 1;85(1):130–8Downes K et al. Blood. 2019 Dec 5;134(23):2082–2091. Alhenc-Gelas M et al. J Thromb Haemost. 2010 Dec;8(12):2718–26Duebgen S et al. Am JClin Pathol. 2012 Feb;137(2):178–84.
Exon 7	c.698G > A	p.(Arg233Lys)	Benign	Tolerated	Polymorphism	rs41267007	Conflicting interpretations of pathogenicity	5.92e-3 (37)	PP2, PP4, BP4	Uncertain significance	Amendola LM et al. Genome Res. 2015 Mar;25(3):305–15.
Intron 8	c.728–20G > A	–	NA	NA	NA	rs78230833	NA	4.36e-3 (7)	PP4, BP7	Likely benign	Simmonds et al. Blood. 1996 Dec 1;88(11):4195–204.
Exon 9	c.913C > T	p.(Gln305*)	NA	NA	Disease causing	rs1395378093	Likely pathogenic	NA	PVS1, PM2, PP4, PP5	Pathogenic	Downes K et al. Blood. 2019 Dec 5;134(23):2082–2091.
Exon 10	c.992C > T	p.(Thr331Ile)	Probably damaging	Affect protein function	Disease causing	NA	NA	NA	PM2, PP2, PP3, PP4	Uncertain significance	Novel
Exon 10	c.1153A > G	p.(Met385Val)	Benign	Tolerated	Polymorphism	rs766423432	NA	1.59e-5 (0)	PM2, PP2, PP4, BP4	Uncertain significance	Not reported
Exon 11	c.1168G > A	p.(Glu390Lys)	Probably damaging	Tolerated	Disease causing	NA	NA	NA	PM2, PP1, PP2, PP4, PP5	Likely pathogenic	Wypasek E et al. Pol Arch Intern Med. 2017 Aug 9;127(7–8):512–523. Andersen BD et al. Thromb Haemost. 2001 Dec;86(6):1392–9.
Exon 11	c.1241T > C	p.(Phe414Ser)	Possibly damaging	Tolerated	Disease causing	NA	NA	NA	PM2, PP2, PP4	Uncertain significance	Novel
Exon 12	c.1351C > T	p.(Arg451*)	NA	NA	Disease causing	rs5017717	Pathogenic/Likely pathogenic	NA	PVS1, PM2, PP1, PP4, PP5	Pathogenic	Downes K et al. Blood. 2019 Dec 5;134(23):2082–2091.

(*Continued*)

 © 2021. The Author(s).

Supplementary Table S1 (*Continued*)

Region	Nucleotide change	Predicted amino acid change	PolyPhen2 HumVar prediction	SIFT prediction	Mutation Taster prediction	dbSNP reference number	ClinVar interpretation	GnomAD allele frequency (*N* homozygotes)	ACMG criteria for classification	ACMG classification	References
											Wypasek E et al. Pol Arch Intern Med. 2017 Aug 9;127(7–8):512–523.
Exon 12	c.1468del	p.(Ile490Leufs*6)	NA	NA	Disease causing	NA	NA	NA	PVS1, PM2, PP1, PP4	Pathogenic	Novel
Exon 13	c.1501T > C	p.(Ser501Pro)	Benign	Tolerated	Disease causing	rs121918472	Conflicting interpretations of pathogenicity	2.02e-3 (1)	PP2, PP4, PP5	Uncertain significance	Wypasek E et al. Pol Arch Intern Med. 2017 Aug 9;127(7–8):512–523. Beau-champ NJ et al. Br J Haematol. 2004 Jun;125(5):647–54.
Exon 13	c.1577T > C	p.(Leu526Ser)	Possibly damaging	Affect protein function	Disease causing	NA	NA	NA	PM2, PP1, PP2, PP3, PP4	Likely pathogenic	Novel

Abbreviations: NA, not available/not applicable; UTR: untranslated region.
Note: Not reported: variant is present in, e.g., ClinVar but not published. Novel: variant is not reported in ClinVar/dbSNP/gnomAD and is not published.

 © 2021. The Author(s).
